# A rare case of *Pseudomonas aeruginosa* bacteremia in a newborn with 58 perforations in the small intestine

**DOI:** 10.1186/s12887-020-02466-2

**Published:** 2021-01-05

**Authors:** Yuanyuan Xu, Danqun Jin, Huan Ye, Youfeng Liang

**Affiliations:** 1 Department of Pediatric Intensive Care Unit, Anhui Provincial Children’s Hospital, Hefei, China; 2 Department of Neurosurgery, Anhui Provincial Children’s Hospital, Hefei, China; 3 Department of Cardiology, First Affiliated Hospital of Anhui Medical University, Hefei, China

**Keywords:** Perforation in small intestine, Newborn, Pseudomonas aeruginosa, Bacteremia

## Abstract

**Background:**

Community-acquired infections of *Pseudomonas aeruginosa (P. aeruginosa)* occur very rarely.

**Case presentation:**

*P. aeruginos* was detected in cultures of venous blood and peritoneal exudate of a newborn with 58 perforations in the small intestine. Intravenous administration of imipenem cilastratin sodium and emergency abdominal surgery were performed. The patient fully recovered and was discharged 17 days after the operation.

**Conclusions:**

Mild symptoms of systemic infections in newborns may delay the diagnosis. Early detection and timely treatment are the key to improved prognosis.

## Background

*Pseudomonas aeruginosa (P. aeruginosa)* is a conditional pathogen commonly found in water, soil, air, on human skin, in the respiratory tract, and intestine. It was first isolated by Gessard in 1882 and then recognized as a pathogen by Charrin in 1890 [1]. *P. aeruginosa* is a ubiquitous pathogen capable of infecting all types of tissues. Most clinical cases are nosocomial infections [2]. Community-acquired infections are rare. This type of infection tends to arise in healthy middle-aged patients. It can have an acute onset, rapid progression, and lead to the development of short-term shock. Herein, we report a single case of extensive perforations in the small intestine associated with neonatal *P. aeruginosa* bacteremia. The newborn underwent surgery and was successfully cured.

## Case presentation

A full-term male infant weighing 3.2 kg was born by spontaneous vaginal delivery to a prima gravid mother without premature rupture of membranes or intrauterine stress. He was exclusively breast-feeded since birth until 27 days of life when he was hospitalized due to 2 days of abdominal distention and vomiting. The patient experienced mild watery diarrhea for 1 day before the onset of abdominal distention and vomiting. Physical examination revealed a low fever, a distended abdomen with the absence of bowel sounds, and slightly cool limbs without delayed capillary filling. Blood tests showed a white blood cell count of 14 780/µL with 81% neutrophils, slight anemia, normal platelet count level, and a markedly elevated CRP concentration of 104.2 mg/L. Abdominal X-ray suggested the presence of free gas under the diaphragm. Intravenous administration of imipenem cilastratin sodium and emergency abdominal surgery were performed.

**Fig. 1:**
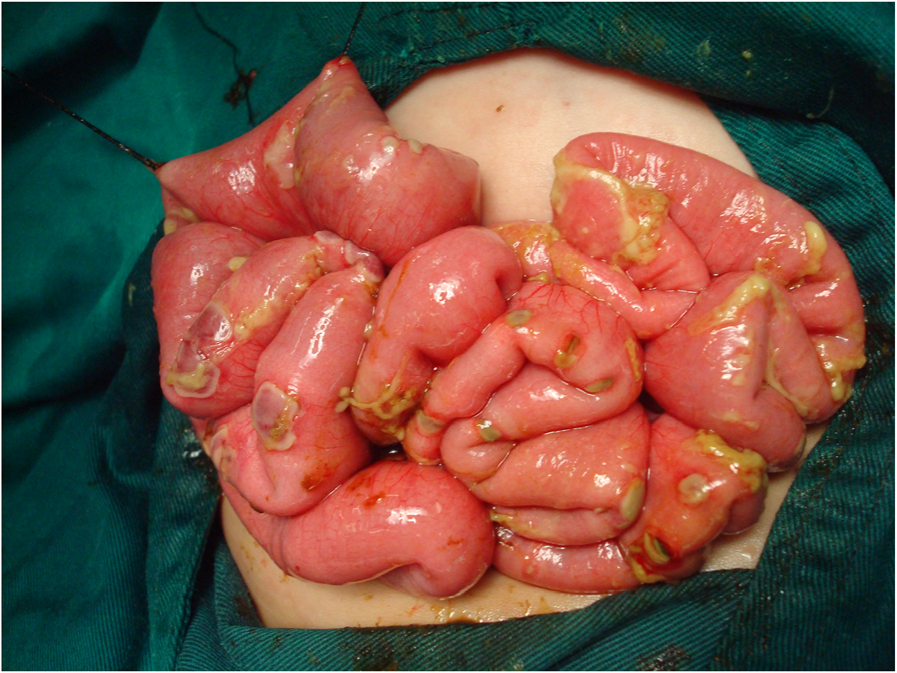
Intraoperative findings revealed multiple focal necrosis with pus and 58 perforations

Intraoperative findings revealed a large amount of yellowish-green cloudy pus, multiple areas of focal necrosis across the entire small intestine, and 58 circular perforations surrounded by blackened, necrotic bowl tissues sitting on red, crater-shaped protrusions (Fig. 1). Wedge resection of necrotic bowel and repair of the perforated sites were immediately performed. Histopathology of the resected small intestine showed a large number of acute and chronic inflammatory cell infiltrations with purulent inflammation in the intestinal wall, muscular congestion and edema, local loss of mucosa with necrosis to the serosa, dilatation of local small blood vessels in the submucosa and massive red blood cells in the stroma near-vessel wall. *P. aeruginosa*, which was sensitive to imipenem, meropenem, and amikacin, was detected in cultures of venous blood and peritoneal exudate.

The post-operative diagnosis was extensive perforations in the small intestine associated with neonatal *P. aeruginosa* bacteremia and acute diffuse peritonitis. The patient showed no postoperative complications, such as intestinal fistula, incision infection, and intestinal adhesions. On the 4th post-operative day, a small amount of feeding was administrated. The patient was discharged on the 17th post-operative day. The baby has been healthy since then, without recurrent infections, malnutrition, intestinal obstruction or other discomforts.

## Discussion and conclusion

This case was characterized by neonatal onset and multiple focal necrosis in the small bowel with 58 perforations. Although the condition rapidly progressed to perforations in the small intestine and peritonitis, the newborn’s symptoms of systemic infection were mild. To the best of our knowledge, this is the first report of extensive perforations in the small intestine associated with neonatal *P. aeruginosa* bacteremia.

Previously, Waldhausen et al. [3] reported that *P. aeruginosa* is the most common etiologic factor of fulminating necrotizing colitis. If the colon was infected, the patient’s symptom would be much more severe or even fatal. The small intestine is a common target organ of *P. aeruginosa* enteric disease. A case with ecthyma gangrenosum combined with multiple perforations of the small intestine associated with *P. aeruginosa* was reported in Japan, where a 13-month-old boy presented with several episodes of watery diarrhea, pyrexia, and irritability [4]. Halder et al. [5] reported a case of a 9-month-old baby girl with *P. aeruginosa* enteric disease who acted irritable since the onset of the fever. An emergency abdominal surgery was performed to repair 5 perforations of the ileum. On 10th post-operative day, the patient succumbed to multiple organ dysfunction syndrome. Presumably, the insignificant symptoms of systemic infection in our case were due to the location of the enteric disease and neonatal onset. There are many bacteria in the colon, which may lead to systemic infection symptoms after colonic perforation. In contrast, after perforation of the small intestine, food and digestive fluid flowing into the abdominal cavity may contribute to the chemical peritonitis. Therefore, early peritoneal irritation is more apparent than infection. Furthermore, the newborn’s immune system is immature, and the early symptoms of neonatal sepsis are often atypical. *P. aeruginosa* can colonize in the gastrointestinal tract. The antigen-presenting cells responsive to bacteria in the normal flora, including *P. aeruginosa*, may be important for maturation of the immune system in newborns [6].

The pathogenicity of *P. aeruginosa* is related to a variety of virulence factors. The PA-I lectin of *P. aeruginosa* may have a key role in its pathogenicity to the intestinal epithelial cells and tight junctions by inducing a permeability defect to its cytotoxic exoproducts such as exotoxin A [7]. Type III toxin can destroy the structure of epithelial cells, resulting in bacteria to pass through epithelial tissue by impaired epithelial barrier function and increased permeability [8]. Therefore, the intestinal colonization of *P. aeruginosa* serves as a reservoir for invasive disease. It can result in ulceration of mucosa and extend into submucosa, causing localized necrosis. Further invasion into muscularis and serosa could cause perforation, peritonitis, and bacteremia [9]. Gross inspection of the intestinal lesions in our case revealed extensive circular perforations with red, crater-shaped protrusions. Histopathology indicated infiltration of a large number of acute and chronic inflammatory cells, with hyperplasia of granulation tissue, inflammatory exudative necrosis, and extravasation of red blood cells, which was consistent with previous reports [4, 5] and highlighted that the intestinal lesions occurred as a consequence of blocked arteries caused by thrombi or bacterial embolization. The patient’s condition was critical when vascular occlusion caused intestinal tissue necrosis, accompanied by infection leading to neutrophil infiltration and abscess formation. Chuang et al. [10] prospectively enrolled 27 consecutive previously healthy children with community-acquired *P. aeruginosa* enteritis and sepsis between July 2003 and June 2012 and found a mortality of 15%.

The apparently mild symptoms of systemic infections in newborns may mislead and delay the diagnosis. Once abdominal symptoms and gangrene lesions of the intestinal tract are observed, *P. aeruginosa* infection should be suspected. Early diagnosis, early administration of antibiotics, prompt surgery, and supportive care are essential for successful treatment.

## Data Availability

Data sharing is not applicable to this article as no datasets were generated or analysed during the current study.
